# Valsalva maneuver, uncontrolled hypertension, asymmetric septal hypertrophy and dynamic outflow obstruction: a case report

**DOI:** 10.1186/1757-1626-2-63

**Published:** 2009-01-19

**Authors:** Ze-Zhou Song

**Affiliations:** 1 Department of Ultrasound, The First Affiliated Hospital, College of Medicine, Zhejiang University, Hangzhou, PR China

## Abstract

A 83-year-old woman presented with a 25-year history of hypertension which was long-standing, uncontrolled, severe hypertension because of irregular oral administration of antihypertensive drug underwent an echocardiographic examination as part of an evaluation of hypertension. She described chest distress associated with activity, syncope for three times in the past one year. On physical examination, she was in no acute distress, with a regular pulse rate and blood pressure of 185/115 mmHg. On auscultation, her lung fields were clear. There was a III/VI late peaking crescendo/decrescendo systolic murmur along the left sternal border radiating to the apex, which increased with standing and Valsalva's maneuver and decreased with squatting. There was no report of provocative maneuvers performed during auscultation. There was no edema. Transthoracic echocardiography showed a hyperdynamic left ventricle with markedly increased left ventricular wall thicknesses and asymmetrical septal hypertrophy. M-mode echocardiography showed systolic anterior motion of the mitral valve apparatus and midsystolic closure of the aortic valve. A dynamic left ventricular outflow tract obstruction was present, with a resting maximal instantaneous gradient of 55 mmHg. With the Valsalva maneuver, the gradient increased to 114 mmHg. No any factors that could cause hypertension were found at kidney, adrenal gland and renal artery etc. by ultrasound and multislice compute tomography.

## Introduction

Hypertrophic cardiomyopathy is the most common disease associated with dynamic left ventricular outflow tract obstruction; however, other conditions may cause similar hemodynamics. Severe uncontrolled hypertension due to pheochromocytoma has been reported to produce severe, and in some cases reversible, left ventricular hypertrophy and left ventricular outflow tract obstruction with surgical removal of the tumor [1,2]. Severe primary and uncontrolled hypertension without any motivation associated with dynamic left ventricular outflow tract obstruction, however, was pretty rare. Here we present a case with uncontrolled primary hypertension, cardiac asymmetric septal hypertrophy, dynamic outflow tract obstruction and increased left ventricular outflow gradients during the Valsalva maneuver in an old woman, which was confirmed by echocardiography, ultrasound, and multislice compute tomography.

## Case presentation

A 83-year-old woman presented with a 25-year history of hypertension which was long-standing, uncontrolled, severe hypertension because of irregular oral administration of antihypertensive drug underwent an echocardiographic examination as part of an evaluation of hypertension. She described chest distress associated with activity, syncope for three times in the past one year. In past, she was never found to have any relevant history of familial heart diseases. On physical examination, she was in no acute distress, with a regular pulse rate and blood pressure of 185/115 mmHg. On auscultation, her lung fields were clear. There was a III/VI late peaking crescendo/decrescendo systolic murmur along the left sternal border radiating to the apex, which increased with standing and Valsalva's maneuver and decreased with squatting. There was no report of provocative maneuvers performed during auscultation. There was no edema.

**Figure 1:**
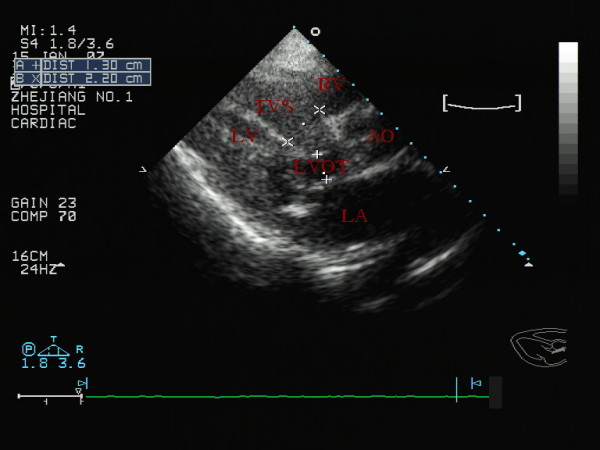
**Left ventricular long-axis view of showing an marked left ventricular wall thicknesses and asymmetrical septal hypertrophy**.

**Figure 2:**
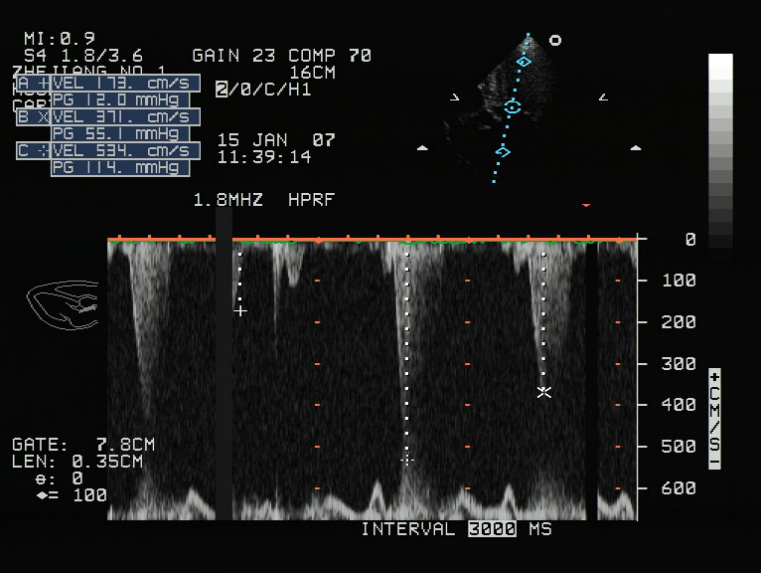
**Left ventricular apical five-chamber view of showing that a dynamic left ventricular outflow tract obstruction was present, with a resting maximal instantaneous gradient of 55 mmHg (B: peak velocity, 3.7 m/s)**. With the Valsalva maneuver, the gradient increased to 114 mmHg (C: peak velocity, 5.3 m/s).

Transthoracic echocardiography showed a hyperdynamic left ventricle with markedly increased left ventricular wall thicknesses and asymmetrical septal hypertrophy (Fig. 1). M-mode echocardiography showed systolic anterior motion of the mitral valve apparatus and midsystolic closure of the aortic valve. A dynamic left ventricular outflow tract obstruction was present, with a resting maximal instantaneous gradient of 55 mmHg (peak velocity, 3.7 m/s). With the Valsalva maneuver, the gradient increased to 114 mmHg (peak velocity, 5.3 m/s) (Fig. 2).

No any factors that could cause hypertension were found at kidney, adrenal gland and renal artery etc. by ultrasound and multislice compute tomography.

## Discussion

The cases demonstrate alterations in left ventricular outflow tract gradient in patients with uncontrolled primary hypertension, cardiac asymmetric septal hypertrophy, dynamic outflow tract obstruction during Valsalva maneuvers. The hypertrophied septum projecting into the left ventricular outflow tract produced high velocity blood flow and resultant anterior motion of the mitral valve, which were deemed responsible for the obstruction. With altered contraction of the septum, a reduction in the displacement of the mitral valve apparatus as well as improved cross-sectional area of the outflow tract was identified. Since the left ventricular outflow tract gradient is dependent on contractility as well as loading conditions, alterations in either of these two variables may produce a variation in the outflow tract gradient in the absence of mechanical intervention. The present case highlights that the Valsalva maneuver decreases left ventricular filling which was the so-called preload, thereby augments the tendency for abnormal mitral valve motion, increased outflow velocities and left ventricular outflow gradients and that long-standing, uncontrolled, severe hypertension could cause cardiac asymmetric septal hypertrophy, dynamic outflow tract obstruction and increased left ventricular outflow gradients during the Valsalva maneuver.

## A list of learning points

1. Uncontrolled Hypertension could also cause Asymmetric Septal Hypertrophy, hypertension control is of very importance.

2. Valsalva Maneuver could induce Dynamic Outflow Obstruction and could substitute the drugs in some cases.

## Competing interests

The author declares that they have no competing interests.

## Consent

Written informed consent was obtained from the patient for publication of this case report and accompanying images. A copy of the written consent is available for review by the Editor-in-Chief of this journal.

## References

[B1] Huddle KR Kalliatakis B Skoularigis J Pheochromocytoma associated with clinical and echocardiographic features simulating hypertrophic obstructive cardiomyopathy Chest 1996 109 1394 1397 10.1378/chest.109.5.1394 8625697

[B2] Lopes HF Silva HB Frimm Cde C Bortolotto LA Belotti G Pileggi F A false diagnosis of hypertrophic myocardiopathy in pheochromocytoma Arq Bras Cardiol 1995 65 167 169 8554495

